# Filming disorganized attachment

**DOI:** 10.1093/screen/hjw042

**Published:** 2016-12-01

**Authors:** Robbie Duschinsky, Sophie Reijman

There are many points of contact between film and psychology. Across the decades, scholars have made film the object of psychological analysis,^1^ while psychiatrists and psychologists pervade cinema and television, from Alfred Kinsey to Hannibal Lecter.^2^ Early documentary films addressing psychology include *Glub Glub and the Monkeys* (Robert Allen, 1975), about the research of Robert and Joan Hinde on infant development in primates. More recent works in this genre are, among others, *The Human Behavior Experiments* (Alex Gibney, 2006), about the Milgram and Zimbardo obedience experiments, and *The Dark Matter of Love* (Sarah McCarthy, 2013), about the family relationships of adopted children who had previously received care in institutions. Yet despite these engagements between psychology and film, the vast archive of film footage *produced* by psychological science has been almost entirely neglected as an object of film studies scholarship. It is true that there has been some commentary on the use of film in science:^3^ in terms specifically of psychological science, Emma Wilson has offered a brief but beautiful analysis of Alain Resnais’s use of footage from evolutionary psychology in *Mon oncle d’Amérique*,^4^ while Lisa Cartwright has studied the films made by René Spitz of infants in institutional care and the role played by these films in scaffolding calls for reform in care arrangements for children.^5^ Apart from these exceptions, however, psychological footage itself has not to date been subject to the tools of film theory. Similarly no attention has been paid to the rich and intriguing methodological discussions about film within the psychological literature itself: regarding the advantages of using silent film as stimulus material to assess children’s attributions about the beliefs and desires of others, for instance, or of using film as a stimulus in neuroimaging research when a subject is unable to move their head.^6^ The absence of attention to these fields is at odds with a growing trend in wider humanities scholarship, in which critics are starting to engage in meaningful dialogue and contention with the sciences rather than tacitly complying with the doctrine of the two inhabiting ‘separate spheres’.^7^ In paying close attention to psychological footage from the field of attachment research, this essay continues the encounter between object-relations theory and film that received recent expression in Annette Kuhn’s *Little Madnesses*,^8^ and also develops attention within film scholarship on the politics of the figure of the child in cinema.^9^

Attachment theory is among the most influential of contemporary developmental paradigms. Bringing work in this area into contact with film theory develops a conversation about film that has long been taking place among psychological researchers. Attachment theorists have, on many occasions, emphasized and discussed the significance of film to their work, and also called for more attention to be paid to the special properties of film media that have facilitated this role.^10^ Indeed, from its very beginning, attachment theory has always been intimately bound up with screen media. John Bowlby, the psychoanalyst and psychiatrist who founded attachment theory, developed his arguments on the basis of a collaboration at the start of the 1950s with James and Joyce Robertson, which involved filming young children who were separated from their parents by hospitalization. Dissemination of the films led to widespread public recognition of the suffering such institutional separation could cause, and contributed to changes in policy regarding visiting opportunities for the parents:

visual portrayal of the grief of one little girl and of her pathetic attempts to control its expression succeeding in convincing countless members of both the hospital and psychiatric professions whom written records had left untouched.^11^

Upon reunion, the children showed signs of disorientation, lack of affection and anger towards their parents, and periods of inconsolable distress. The psychological significance of the separation was understood to be *visible* as such on the screen to viewers of these films. Commenting at the time, Anna Freud described the film technology used by Bowlby and the Robertsons as remarkable for its capacity to make ‘the outward manifestations of the inner processes’ available for demonstration and analysis.^12^ Such comments by psychologists in the 1950s can be regarded as echoing the magnetic interest of the late nineteenth-century ‘Child Pictures’, which captivated audiences with their capacity to make the authentic and inner animations of a child’s feelings visible and mass-reproducible.^13^

At the heart of attachment theory lies Bowlby’s proposition that the primate infant continually monitors the availability of at least one determinate caregiver (their ‘attachment figure’), who is anticipated to be able to offer protection and support. When a familiar caregiver appears to be physically and attentionally available and the infant is calm, the child can engage in activities such as play or sleep; after a period without interaction, but especially when alarmed or anxious, the infant will cease other engagements and seek the proximity and availability of her attachment figure through behaviours such as crying, smiling or crawling. Bowlby termed this process the ‘attachment behavioural system’, and proposed that it evolved to facilitate the child’s survival in the face of dangers such as predation, attack or exposure to the elements. Bowlby’s ideas were tested and extended by North American developmental psychologists, who went on to utilize film to document individual differences in behaviour shown when the attachment system is activated. The most influential such assessment was the Strange Situation Procedure, an observational measure designed by Mary Ainsworth in the mid 1960s. In this experimental procedure, as the episodes increase the infant’s sense of alarm by increments, the observer can attend to individual differences in the infant’s movement between behavioural systems: the interplay of exploration of toys and attachment behaviour, in the presence and in the absence of the parent and of a stranger. The procedure lasts for twenty minutes; the reason being, as Ainsworth recalled in interview, that this was the amount of time that would fit easily on one reel of 16mm film, the standard medium of the period.^14^ In a letter to Bowlby, Ainsworth enthused about her laboratory’s access to an ‘excellent […] portable videotape apparatus’, which could be used to record the Strange Situation from behind a one-way mirror. The capacity to repeatedly review infants’ and parents’ gestures on tape, and to watch them in slow motion, Ainsworth wrote, was simply ‘beautiful’.^15^

In their ‘instructions to camera operators’, Ainsworth and her students specified that a wide shot should be used to capture caregiver and child in frame together on their entry and exit from the Strange Situation and at times when they interact and play. For moments when the child is playing alone, or experiencing the departure or return of their attachment figure, ‘close-ups are essential’.^16^ In general, though, it was advised that

focusing on details of facial expression and how toys are manipulated is not helpful for scoring. Posture and movements of the arms and legs (e.g. kicking, stiffening, pushing or leaning away) are critically important and will be lost if the camera operator is too fond of close-ups.^17^

Accordingly, throughout an observation the camera generally focuses on the child’s body and attends particularly to their communication with their attachment figure; it is seen as good practice, when filming a Strange Situation, to keep the child’s face towards the top of the shot and their torso to the centre, allowing the camera to ‘fill the screen with the codable image’.^18^ In her published work, Ainsworth herself praised film technology in particular for its capacity to support ‘finer-grained’ analysis of the different and conflicting motivations that might be read off from the morphology of a child’s posture or gestures, allowing the observer to use visible behaviour to infer the interplay between behavioural systems such as exploration and attachment.^19^ The camera can make visible – in the movement of knees, lips and fingers – all kinds of half-gestures, mumbled vocalizations and fleeting affects, suggestive of emotional dynamism and the complexity of distress and desire. It allowed attachment theory to be recognized as a form of developmental science, on the basis that the ‘analysis of video materials is replicable and it can be checked’.^20^

Beyond academia, films tracking infant attachment behaviour have been used by clinical and social welfare professionals, and have been drawn upon in risk assessments of child–caregiver relationships. There is also an extensive and growing use of filmed observations of children’s attachment behaviour in feedback given by professionals to caregivers in circumstances where help has been requested or concerns raised about maltreatment.^21^ For instance, courts or child welfare authorities may mandate video-feedback support from professionals after children have been returned to their caregivers after a crisis and a period in foster care. Attachment researchers and clinicians have argued that ‘the effects of the experience of watching oneself on video-film, especially when watching oneself interacting with one’s child, can be startling and evocative in ways that promote change. Multiple sensations, emotions, beliefs and representations are aroused, often in unsettling ways, very likely activating the attachment system’ in the parent, and making attachment representations available for discussion and change.^22^ Moss and colleagues, describing how the qualities of film specifically enable such an intervention, state:

Video-feedback offers an opportunity to provide immediate feedback on caregiver–child experiences which have just occurred during the 10–15 minute filmed interactive period. This is in marked contrast to typical clinical models where events are often discussed after considerable delay. The immediacy of the therapeutic experience and the use of video sequences of the caregiver and child also allows for easier access to self and other cognitive/affective representations for purposes of change. Viewing one’s own behavior, while being guided by a sensitive intervenor, may help the parent rework distorted representations, either overly negative or idealized, that maltreating parents often have of themselves or the child.^23^

Professionals report using particular sequences of film as a route towards grounded, non-accusatory conversations about parents’ typical ways of responding to their child, as well as consideration of alternative strategies for showing love, regulating anger and anxiety, and having fun together. When successful moments of interaction are highlighted, alongside the actions that brought about these moments, parents can be helped to find an image of themselves on film as a model for their own future exchanges with their child.^24^

Thousands upon thousands of films tracking the interrelation of infant distress, play and attachment behaviour have been made by psychological researchers and psy-discipline professionals. In particular, the Strange Situation has confronted infants with two separations from and reunions with their caregiver, with a camera recording their response to these alarming episodes and the extent of their distress. The increasing availability and affordability of filming equipment has added impetus to the films’ production; this accumulation of recordings inevitably warrants interrogation, along the lines of Vicky Lebeau’s enquiry, ‘What wishes are at work in the visual archive of the child in pain?’^25^ Lebeau’s incisive and difficult question, in its original context, was directed at the role of the image of the child as the privileged exemplar used by early cinema to demonstrate its capacity to capture authenticity in the affects on screen and evoked in viewers. Yet it has a bearing on films of infant attachment, most notably those of the Strange Situation, since here the filmic image of the suffering child is reinvested as the mark of species-wide abilities, developed in infancy, to regulate affect in ways responsive to the earliest caregiving environment, and anticipated to have developmental consequences for later happiness and relationships.^26^

Lebeau has explored early cinema’s investment in the image of the child as testament to its authenticity in capturing feeling, and Cartwright has considered the moral authority given to psychologists by films of suffering infants. Our work here is influenced by theirs. Focusing specifically on the research psychology context, it will show how viewers of filmed Strange Situations have ‘completed’ the films they are watching, and how they have invested in visual technology as an enhanced way of making meaning of continuities and discontinuities in infant gestures and movements.

Three categories were originally proposed in 1969 by Ainsworth and her assistant Barbara Wittig for coding patterns of infant responses to their Strange Situation Procedure. In films classified as ‘avoidant’ (Group A), the infants avoid the caregiver with their gaze and posture when reunited. Although these infants seem poised and unruffled, studies of heart-rate and cortisol show that they are actually quite distressed by the separation. Home observations reveal that avoidance on reunion in the Strange Situation represents a way that the infants can keep their distress from becoming visible to a caregiver known to be likely to reject expressions of a desire for comfort.^27^

In films classified as ‘secure’ (Group B), the infants display more elaborate play, use the caregiver as a safe base from which to explore the toys and a safe haven to which they may return, especially when alarmed or anxious. These infants are visibly distressed at their caregiver’s departure – but on the second reunion (and perhaps the first as well) look to the caregiver’s face, show pleasure, seek the caregiver upon his or her return, and can be comforted and return to play within three minutes. Based on home observations of these infants and caregivers, it was theorized that the infants respond as they do in the Strange Situation because they feel confident that their communications about need can get through to their caregiver and that the attachment figure will be available to protect and support them when called upon.

Children classified as ‘ambivalent/resistant’ (Group C) show distress even before separation and are clingy and difficult to comfort on the caregiver’s return. They distrust their caregiver’s offer of toys to play with, treating such offers as attempts by the caregiver to distract them from receiving attention and affection. Home observations revealed that the caregivers of these infants were not contingent in their responses to their infants’ signals. The infants were theorized to be pre-empting their caregiver, deploying distress and anger to get and keep the attention and availability of a caregiver who was not expected to be predictably responsive.

The A, B and C patterns of attachment were conceptualized by Ainsworth as ‘organized’. Their technical understanding of the term deviated from its use in everyday language, instead meaning that the attachment system could recruit and orchestrate a variety of behaviours (for example, crying, smiling or crawling) to achieve some form of protective proximity with their caregiver.^28^ The secure (B) pattern is understood to achieve the physical and attentional availability of the caregiver directly, by communication of distress and acceptance of comfort; the avoidant (A) and resistant (C) patterns achieve this availability in a ‘conditional’ way, downplaying their distress or displaying it intensely and without apparent trust in their caregiver’s overtures.

The attachment classification the infant received with one parent was found to have no association with their classification with the other parent, implying that the Strange Situation coding protocols to a large extent do not assess personality differences but relate to the infant’s expectations about the care they will receive from a particular attachment figure. The infant classifications were found to be predictive, to some degree, of all manner of later assessments of mental health and of social and academic competence.^29^ Researchers concluded that the Strange Situation assessment and classifications were tapping a foundational aspect of the way that an infant’s early caregiving environment influences their development. The direct association of infant attachment classification with later outcomes was not generally found to be high except on a few specific measures, but the cumulative indirect effect was surprisingly substantial.^30^ That the same patterns of infant behaviour could be found by researchers time and again, with relative consistency across contexts and cultures, provided evidence both that human infants have a predisposition to seek their familiar adult when anxious and that this predisposition can be moderated, if necessary, in two ways: by downplaying distress in an avoidant pattern in order to keep self-control and avoid rebuff, or by pre-empting an unpredictable caregiver by using distress and anger as a way of keeping the caregiver’s attention. The three Ainsworth attachment patterns were thus understood to be such robust constructs because they were the product of *universal capacities of human emotion-regulation* within early relationships.

It is worth highlighting the universalizing stakes of these discourses because, from the 1970s, an archive began to grow of filmed recordings in which children, particularly on reunion with their caregiver, displayed gestures and postures *discrepant* with Ainsworth’s coding system. There were films from the research of Ainsworth’s doctoral students in which the infant ‘appears to be “in a trance”’, for example, or in which an infant ‘slaps his own face; pulls his ear; digs into his arm with his nails’, or displays ‘odd vocalisations (he “barks”)’.^31^ Infants were also observed tensely cocking their heads,^32^ or throwing their hands in front of their faces on reunion with their parent. Many researchers in the 1970s concluded that these gestures and postures were mere ‘noise’, produced by an inability of the Ainsworth Strange Situation procedure to cleanly draw out the attachment system for filmed observation – children do weird things. However, the films in which these discrepant behaviours were visible were more common in samples taken from children under clinical or social service care or in otherwise at-risk populations. And the potential meaningfulness of this archive of discrepant films was corroborated by observations from child psychoanalysts, such as Selma Fraiberg, who saw in their clinics just such behaviours interrupting the play of infants who had experienced trauma or neglect.^33^ In 1981 Mary Main introduced a new ‘unclassifiable’ designation for such films, with the hope that the new (non-)classification would both improve the validity of the Ainsworth ‘secure’ classification by removing children who approached the caregiver on reunion, but did so in conjunction with discrepant behaviours suggesting stress or tension. It was also hoped that the identification of these discrepant cases and their consideration together might itself turn out to be fruitful. The indelible debt that this development owed to film technology was emphasized by Main and her colleagues: ‘the distinction between secure and unclassifiable, false-secure infants has been made possible only by the opportunity for close, repeated review of videotape records’.^34^

As film theorists such as Mark Hansen and Laura Mulvey have discussed, channeling representations of movement on film through repetition, slow motion and freeze frame can offer important new possibilities for seeing.^35^ In the case of attachment research, film technology facilitated researchers’ repeated viewing of the same behaviour, paying attention to what came before and after, and its particular physical morphology and apparent social significance in the dance of interaction between child and parent. For example, in one Strange Situation filmed in Main’s laboratory, an infant interrupted her approach to her father on reunion. ‘She suddenly stopped and turned her head to the side and – while gazing blankly at the wall – slapped a toy and then her empty hand on the floor’, before continuing her approach and reaching to be picked up. The researchers found out afterwards that the child’s father had recently attempted suicide and had frequent homicidal fantasies.^36^ Main and colleagues noted that in such cases ‘slow-motion review of the tape yielded a strikingly different interpretation’ of gestures and postures that were ‘too subtle to note in real time’, and might otherwise have been dismissed as mere ‘noise’.^37^ In an interview, Main and Erik Hesse recall

observing a startling asymmetrical smile in the first three seconds of reunion with the mother, where one side of the mouth turned up as in a smile, the other down as in a grimace. While the mouth movements vanished almost immediately, they were verified in slow motion. Later, it was learned that the baby had been maltreated.^38^

Describing the infant of this film in print, Main and Judith Solomon describe that ‘in these microseconds, her eyes widen as she looks at mother [and] the asymmetry makes her appear puzzled, disgusted or fearful. Her face then breaks into an extremely wide smile’.^39^

The interruptive quality of such movements and expressions would not have been available for scrutiny if not for the use of film. Perhaps, however, the greatest significance of film for research on these discrepant behaviours lay in the fact that it allowed researchers to confer. That films could be shown to and discussed with others meant that behaviours that would otherwise have been tacitly disregarded could be treated as potentially meaningful. Main’s laboratory at Berkeley began to collect unclassifiable tapes from other researchers working with high-risk samples, such as Mary J. O’Connor, Elizabeth Carlson, Patricia Crittenden and Susan Spieker. A member of the laboratory, Solomon, conducted an intensive review of tapes of unclassifiable behaviour in infant–parent interactions, with a particular focus on sixty tapes that included infants from both low-risk and high-risk samples. Solomon observed a variety of anomalous behaviours that were particularly common in the maltreated sample: apparent signs of depression in infants; indications that an infant was attempting to muster an A, B or C pattern but failing to achieve this; infants initially approaching the caregiver but then veering off; disoriented behaviours (such as the child leaving its arm hanging in the air).

In their publication suggesting that these anomalous tapes point to the need for a new attachment classification, Main and Solomon reported two overriding themes:

the most striking theme running through the list of recorded behaviours was that of *disorganization*, or, very briefly, an observed contradiction in movement pattern, corresponding to an inferred contradiction in intention or plan. The term *disorientation* was also needed, because, for example, immobilised behaviour accompanied by a dazed expression is not so much disorganized as seemingly signalling a lack of orientation to the immediate environment.^40^

In line with Ainsworth’s technical use of the term ‘organized’, mentioned above, Main and Solomon’s use of the term ‘disorganization’ diverged from its everyday meaning. It was used instead in a technical sense to mean the absence or disruption of a coherent sequence of (visible) behaviours oriented to achieve proximity with the caregiver, presumed therefore to suggest contradiction or disturbance of the (invisible) attachment system itself.^41^

Film technology made it possible for the field to come to a certain degree of consensus about the need for a new classification in addition to Ainsworth’s three categories of infant reunion behaviour. At a four-day workshop at the University of Washington in 1985, Ainsworth sat on the floor to be as close as possible to the screen while Main showed her tapes coded with the new ‘D’ classification; at the end of the event, Ainsworth wrote to Bowlby that she and ‘everyone there was most impressed with the need for adding a new “D” or disorganised category to the classification system’.^42^ Subsequent research has found that infants who display a significant degree of behaviour discrepant with the Ainsworth categories are at elevated risk of social and mental health problems as they grow up. For instance, such infants have been found to be more likely to show aggressive and violent behaviour later in life;^43^ and a classification of disorganized/disoriented attachment in infancy predicts the severity of PTSD symptoms following a trauma – an association that was found not to be attributable to the many concurrent risk factors.^44^

In general, recordings of Strange Situation Procedures that have been assigned the ‘disorganized/disoriented’ (D) classification are not accessible to the public, in order to preserve the confidentiality of the participants. However, anonymized extracts without sound from two such videos have been made available online by researchers from the SUNY Stony Brook and the New York Attachment Consortium.^45^ In one of the two extracted videos we see an infant during a separation from his mother. He is in the centre of the frame, holding his finger out to touch a yellow duck that has been presented by a stranger, who is partly offscreen. The stranger appears pleased to be interacting; the infant’s face is flat, almost weary. The door opens, and his mother enters the room and pauses briefly; the shot captures her feet, neatly together but not directed towards the child, as she presumably regards him. Without changing his flat expression, the infant looks up towards his mother’s face but does not otherwise greet her. The camera moves the stranger out of shot, except for the edge of her black shoes. As the mother returns to her seat, the child turns away and shifts from a sitting position to pitch forward – as if in genuflection – diagonally away from his mother’s movement past him. He lies prone with his cheek against the carpet, facing towards the stranger; his mother cannot see his face from this position. His fingers are splayed on the floor, his mouth is open and his eyes are wide: it is not clear whether this is a look of surprise, pleasure or horror. It could be all three. Then his eyes close towards slits. With his mother now in her chair behind him, the infant uses his arm to right himself, his mouth opening yet wider. Upright and with one leg tucked under his body, the little boy’s face is relaxed and he moves expansively to collect a ball that is outside his immediate reach. He holds the ball to his chest and looks up at his mother. His eyes are a little wide and his lower lip is slack; he appears worried. Then, with a slight, friendly smile, he holds the ball out to his mother and looks into her face. He throws the ball towards her, seeming pleased and a little cheeky.

The disparity in affect between this apparently happy play and the genuflection away from the mother on her return to the room moments before is stark and disconcerting to a viewer familiar with infant behaviour on reunion with their caregiver. Without the capacity of film to intensify our attention to particular scenes of movement and affect it would be difficult even to identify this fleeting disjuncture, and probably impossible to generate any consensus between coders about what it might mean in attachment terms. At a behavioural level the infant’s behaviour sequence could be considered as a kind of avoidance (A), since the infant is attempting to mask his distress from the parent and is directing his attention away from her; it is classed also as ‘disorganized/disoriented’ (D), because coders can infer a disruption of coherence of the attachment system in the context of countervailing affects.

As we have seen, ‘disorganization’ was defined by Main and her colleagues as an observed contradiction in movement pattern, understood to reflect a parallel disruption of the infant’s representations of the caregiver that integrate the attachment system. Main clustered such behaviours into seven indices, which could be used by researchers to code ‘attachment disorganization/disorientation’. Main and Hesse proposed that these diverse forms of behaviour could all be explained in terms of a contradiction between the attachment system and another behavioural tendency. Reflecting on what tendencies could be sufficiently powerful to disturb the attachment system, which demands that the infant seek protection from the attachment figure when alarmed, Main and Hesse concluded that it must be alarm evoked by the attachment figure themselves. That is, ‘an infant who is frightened by the attachment figure is presented with a paradoxical problem – namely, an attachment figure who is at once the source of and the solution to its alarm’.^46^ A parent who frightens the child with abusive behaviour, or who themselves is frightened when the child seeks comfort because of past trauma, could both be supposed to cause such a paradox for an infant. In their later work Main and Hesse, alongside other researchers such as Solomon and Carol George, emphasized the disorganizing role of deferential, helpless or withdrawing behaviour displayed by a parent to their infant. Such behaviours do not proximately frighten the child; however, if an attachment figure withdraws when their infant looks to them for protection and support in regulating their feelings, this may lead to experiences of unassuaged alarm, which ultimately become frightening for the infant.^47^

Looking back, Main has claimed that her ideas have been mischaracterized in a manner ‘widespread and dangerous’,^48^ which has contributed to the misuse of the concept of disorganized/disoriented attachment by clinicians and social workers in screening for maltreatment.^49^ Main and Hesse, acknowledging that the emphasis of earlier papers may have misled readers, have subsequently stated that they wish they had made it clearer that their emphasis on frightened or frightening caregiver behaviour was ‘one highly specific and sufficient, but not necessary, pathway to D attachment status’.^50^ They draw attention to their own research findings that show that unresolved bereavement in a parent who is otherwise caring safely and well for their child predicts a classification of disorganized/disoriented attachment in the Strange Situation. They also draw attention to other studies, which highlight that a parent’s ongoing experience of an anxiety disorder or multiple forms of social and economic disadvantage have been found to predict disorganized attachment in the infant – even in the absence of any known maltreatment or neglect.

Looking in retrospect at Main’s texts of the 1990s from the vantage of the present, it is clear that mischaracterizations of disorganization/disorientation were facilitated by the predominant narrative of her writings, which gave the impression that she was proposing a unitary maltreatment category within an exhaustive taxonomy of child behaviour, even though qualifications are clearly there in the texts if looked for.^51^ Yet if ‘disorganized/disoriented attachment’ was a concept erected upon film technologies, it can be suggested that the concept was also in part embalmed by the way film was used and understood. Whether drawing upon hermeneutic phenomenology or psychoanalysis, since the late 1970s film theorists have drawn attention to the role of the spectator in ‘completing’ the images and sounds they experience to form the meanings of a film.^52^ Main and Solomon precisely and necessarily depended upon this process, since they required viewers to *infer* some form of contradiction or disruption of the (invisible) attachment system from the observation of (visible) infant behaviour that did not appear to the viewer to constitute a coherent strategy for proximity-seeking. The term ‘disorganization/disorientation’ was introduced as an imperfect but best available description of the kinds of gestures and postures from which this contradiction or disruption could most directly be inferred.

Coding disorganization/disorientation requires what Goffman terms a ‘frame-break’: an ejection of the viewer from his or her expected involvement and the meanings they expected to find in a sequence of filmed behaviour.^53^ Indeed, the disruption of the expected behavioural sequence represented by disorganized attachment has been described by Alan Sroufe, in training researchers how to code the Strange Situation, as ‘like a video which keeps flickering’.^54^ Both the malfunction of film technology and a visible disturbance of the coherence of an infant’s behavioural sequence on reunion with their caregiver disrupt our expectations regarding genres of motion as the basis for ready intelligibility. There is an integral logic to Sroufe’s analogy, founded on the specific configuration within which screen media releases expectation and attention. Main and Solomon sought to utilize the distinctive and critical capacity of film to throw a viewer back upon his or her assumptions: the coder was expected to watch, rewatch and watch again, considering and negotiating with their assumptions about what a coherent infant strategy for direct or conditional proximity-seeking on reunion might look like. The viewer was enjoined to look for visible clues (such as freezing, or a parabolic approach trajectory to the caregiver on reunion) to indicate that the coherence of the attachment behavioural system was being disrupted or contradicted. This is why Main and Solomon urge that ‘the observation and recording of D behaviour can only be made in conjunction with repeated, slow-motion study of the film’.^55^ It is indicative that Main and Solomon specify that a stationary camera mounted in one corner of the Strange Situation room is sufficient to code a child with an A, B or C Ainsworth classification, but is *not* adequate for making a D coding: it will miss too many of the clues of disruption or contradiction of the attachment system that may be embedded in ‘infant facial expressions and small motor movements’.^56^

However, the immediacy of the vivid and deep representation of events offered by film supported the impression that the meaning of the behaviours was immediately accessible, that viewers could readily *see* disorganization itself as an attachment type expressed within the diverse behaviours – what, in Elizabeth Cowie’s terms, might be considered a ‘desire for the real joined together with the science of the visible’ for the film’s viewer.^57^ This potential to overstate the epistemological sureties of film was supported by the ambiguity of language and narrative in the chapters by Main and Solomon introducing the concept. Yet further fuel towards reification was probably added by the general tendency in psychology to treat behaviours in a group as occurring through a single process,^58^ and by the desire of clinicians and social workers for a classification stabilized as signifying risk.^59^ As a result of this reification of the concept of disorganization, many researchers and ‘psy-discipline’ practitioners took from the work of Main and Solomon the (partially circular) conclusion that all behaviour discrepant with the Ainsworth A, B and C classifications manifest, in an undifferentiated way, a unitary processes of ‘disorganization’.

In their chapter announcing the protocols for coding disorganization/disorientation, Main and Solomon themselves warn that treating the items in a group as expressions of an essence tends to offer undue support to the belief that all phenomena in this group have a single historical cause.^60^ And‘ indeed, perversely, as if this warning had been a prediction, perceptions of disorganization/disorientation as a unitary process have facilitated the widespread misapprehension that all behaviour in the D indices is necessarily caused by an immediate conflict between attachment and fear. This assumption that the indices of disorganization/disorientation express a single process has contributed to a neglect of *how*, through what proximal processes, the indexed behaviours occur. This is a neglect compounded, but not solely caused by, the existence of practical difficulties of statistical analyses, such as the fact that behaviours which index disorganization/disorientation often co-occur in high-risk samples; it would have been possible to circumvent this by coding the Main and Solomon indices using a small number of dimensional scales. As Karlen Lyons-Ruth et al. have recently observed, with concern, ‘to date, few hypotheses have been advanced regarding the mechanisms underlying this striking difference among infants who display disorganized behavior’.^61^ The researchers note that this inattention to mechanisms may be masking important differences and potentially limiting the precision of clinical and welfare interventions. For example, they report intriguing findings that show where disorganized/disoriented behaviour is displayed by a infant without any avoidance or resistance, this has a distinct association with suicidal feelings arising in the subject by the age of nineteen.

For decades attachment researchers have published discussions of the ‘notoriously complex origins of attachment disorganization’,^62^ without considering that some of this complexity may be a product of the mummification of the classification. As Bryan Turner has shown, in unduly subsuming causally important heterogeneity, reification generally ‘causes particular problems for the development of theory regarding the causes and consequences of phenomena grouped together under the label’.^63^ Many basic attachment researchers and professionals deploying ideas from attachment theory have presumed that the only quality that can be ascribed to infant behaviours in the Strange Situation that diverge from the Ainsworth protocols is that they *lack* organization.

An alternative approach is, however, offered by Gilles Deleuze, who argues that we should view all behaviours as the product of aligned or contradictory machines of movement and desire, and classificatory systems as epiphenomenal overlay. Deleuze’s ideas offer a way of sidestepping the conventional assumption that a category of behaviour reflects a single unitary process. Moreover, his film theory offers tools for conceptualizing anomalous forms of movement or gesture, by not reducing these too quickly into unitary categories. Bringing Deleuzian film theory together with developmental science may seem an odd and disjunctive synthesis. In agreement with feminist critiques of psychological discourses,^64^ Deleuze (and his collaborator Fe´lix Guattari) are generally hostile to developmental psychology and its role in normalizing subjectivities through the policing of families. Yet, curiously, they not only hold back from criticism but *specifically* affirm the validity and significance of attachment phenomena: ‘It is not a question of denying the vital importance of parents or the love attachment of children to their mothers and fathers’.^65^

Where it is the case that the attachment system can achieve its centrifugal goal directly (B), or indirectly and conditionally (A and C), the film of a Strange Situation can present what Deleuze calls a ‘movement-image’: a circuit of images in which movement is brought back through a sequence of actions into the arms of an apparently stable and pre-existing order of figures and concepts. A movement-image implies in its resolution that centrifugal movements of exploration are matched by centripetal movements of return, and separation of child from caregiver is merely the interim between states of being together as sufficiency. Such images of natural sufficiency between mother and infant have been deployed as rhetorical ammunition for conservative political demands that precisely isolate women from the health, social or political resources required for sufficiency.^66^

Deleuze defines the movement-image in film as one in which

the sensory-motor schema is concretely located in a ‘hodological space’ (Kurt Lewin), which is defined by a field of forces, oppositions and tensions between these forces, resolutions of these tensions according to the distribution of goals, obstacles, means, detours. The corresponding abstract form is Euclidean space, because this is the setting in which tensions are resolved according to a principle of economy. 67

In hodological space, where ‘opposing forces are equal in strength, their resultant force = 0’.^68^ Deleuze emphasizes that ‘anomalies of movement’ within this principle of economy are only recognized as a single undifferentiated cluster: *disordered*, and as such either ephemeral and meaningless, or in need of rectification.^69^ Yet, for Deleuze, this characterization of anomalies of movement as undifferentiated disorder is a product of a quality of commitment to existing categories, resulting in a failure to recognize that these anomalous movements may have a logic. Glossing Henri Bergson, Deleuze argues that ‘the idea of disorder appears when, instead of seeing that there are two or more irreducible orders, we retain only a general idea of order that we confine ourselves to opposing to disorder’.^70^ In the case of disorganized attachment, researchers and clinicians have confined themselves to opposing Ainsworth’s patterns of attachment with an idea of ‘disorganization’ conceived of as chaotic, meaningless behaviours, unworthy of further investigation for the kind or quality of affect or behaviour in play.

Deleuze contrasts the movement-image to the ‘time-image’, a film in which actions cannot be unified with resolutions. For instance, in the time-image, ‘movement can tend to zero, the character, or the shot itself, remain immobile’, threatening to turn the transitions and transactions of a film into a still photograph through the frozen state of a body:

movement may also be exaggerated, be incessant, become a world movement, a Brownian movement, a trampling, a to-and-fro, a multiplicity of movements on different scales. What is important is that the anomalies of movement become the essential point instead of being accidental or contingent.^71^

In examining the logic of such anomalous movement within film, Deleuze conceptualizes the space of the time-image with the idea of *fluctuatio animi*, borrowed from Spinoza. For Spinoza, *fluctuationes animi* are the counterpart process in the domain of affect and feelings, of confusion and doubt in the domain of the imagination:

For the most part, [*fluctuationes animi*] arise from an object which is the efficient cause of each affect. For the human body is composed of a great many individuals of different natures, and so it can be affected in a great many different ways by one and the same body. And on the other hand, because one and the same thing can be affected in many ways, it will also be able to affect one and the same part of the body in many different ways. From this we can easily conceive that one and the same object can be the cause of many and contrary affects.^72^

*Fluctuatio animi* thus occurs to the degree that the complexity of dynamic forces within which the subject is situated, and of which she is composed, inhibit the smooth alignment between affect and a behavioural sequence.^73^

The *fluctuatio animi* of the time-image, Deleuze concludes, ‘is not hesitation between several objects or between several directions, but a mobile covering-up of sets which are incompatible’.^74^ It is an ‘undecidibility of the body’, in which ‘the obstacle does not, as in the action-image, allow itself to be determined in relation to goals and means which would unify the set’.^75^ As such, the time-image is not evoked by contradiction in the abstract, but by the concrete possibility of *imperatives for gestures of the body, which are incompatible within a behavioural sequence*. This potential incompatibility of behaviour, suggestive of incompatibility at the level of affective intensity, allows the film of infant behaviour in the Ainsworth Strange Situation to be regarded as a time-image: it reveals a world in which not everything is ultimately reconcilable with full coherency, with a consequence in behavioural and relational instability.

Main’s emphasis in conceptualizing disorganized/disoriented attachment is the role of fear; although, contrary to common misconception, she does not assume that fear in relation to the caregiver is always the proximate cause. Treating disorganization/disorientation as a time-image raises the question of other proximal causes of *fluctuatio animi*. This is a concern that has existed largely outside of any awareness of attachment theory, and which represents a contribution to psychological theory on offer in scholarship on film. Asking, with Deleuze, about the causes of *fluctuatio animi* directs our attention to the sizeable segment of behaviours that are used to code disorganized/disoriented attachment but *do not* suggest an immediate conflict between attachment and fear as their proximal cause. Some indicate a child flooded by distress. Others suggest anxiety and tension. Yet others, however, appear to be the result of a conflict between attachment and anger. For instance, in the Main and Solomon coding protocols, Index II directs attention to occasions when ‘the infant displays anger simultaneously with proximity seeking or contact maintaining’; and Index III suggests that disorganization is coded when an ‘infant interrupts approach to parent on reunion with a bout of angry behaviour, directed away from the parent, then continues approach’.^76^

A testable hypothesis for attachment researchers emerging from such a consideration is that *fluctuatio animi*, suggesting a conflict between attachment and fear, might have different precursors and sequelae than behaviour suggesting a conflict between attachment and anger/frustration. Hundreds of studies of infant attachment in the Strange Situation will have had access to the data to test this hypothesis, since every child must be assessed for anger on a one-to-seven scale according to Ainsworth’s basic coding protocols. To date, however, the matter has been excluded from intelligible ways of thinking about disorganization. Researchers have neither discussed nor considered how anger and the behaviours identified by Main and Solomon may co-vary. In this vacuum of inquiry and thought, some strange decisions have been made by researchers: some have treated angry behaviours by children as if they all represented disorganization; others have treated all the behaviours used to code disorganization as if they must really all indicate anger.^77^ Are attachment/anger conflicts the same as attachment/fear conflicts? This question remains not only unanswered but *unasked* by attachment researchers and clinicians drawing on the concept of disorganized attachment, yet it represents an example of how thinking with Deleuzean film theory can speak back to psychological theory. As such, the claim of this essay is not only that film theory can be applied productively to developmental psychological footage, but that doing so offers benefits to both disciplines. Film theory has the potential to offer psychology tools for considering more finely the relationship between behaviour and interpretation, and for tracing different flows of movement and affect, for which attachment categories are an epiphenomenal overlay in the classification of infant–caregiver reunions in the Strange Situation.

We have, in this essay, considered how the influential ‘disorganized/disoriented’ (D) attachment classification first emerged as a consequence of film technologies and has subsequently been reified and limited by the ways in which these media have been interpreted. After examining the relationship between screen media and the development of the attachment classification, we have drawn upon ideas from Deleuze’s theory of cinema in interrogating the concept of attachment disorganization/disorientation. Where disorganization/disorientation is conceptualized within the hodological space of the movement-image it appears merely as chaos and disorder, since in hodological space, where ‘opposing forces are equal in strength, their resultant force = 0’.^78^ In infant behaviour in the Strange Situation, considered as a movement-image, reality is simplified to a stark and false opposition: either there is smoothly coordinated movement towards the telos of the attachment system in the full (B) or conditional (A or C) availability of the caregiver, or there is nothingness and/or undifferentiated pathology. Yet considering disorganized/disoriented attachment as a time-image draws attention to its logic as *fluctuatio animi*, a physical irreconcilability of intense affect, within infant behaviour on reunion with a caregiver. Such an account does not quickly dismiss disorganized/disoriented attachment; it does, however, countermand tendencies towards its ossification and misapplication. It also opens a new avenue for thought and empirical study in developmental psychology. Deleuze’s film theory suggests, for example, that conflict between attachment and anger may warrant further scrutiny from researchers.

In the *Archaeology of Knowledge*, Foucault observes that:

a discursive formation does not occupy […] all the possible volume that is opened up to it by right of the systems of formation of its objects, its enunciations, and its concepts; it is essentially incomplete, owing to the system of formation of its strategic choices. Hence the fact that, taken up again, placed, and interpreted in a new constellation, a given discursive formation may reveal new possibilities.^79^

Our intention here has been both to place film theory in a new constellation, in demonstrating its value for thinking about filmed psychological assessment as a different kind of object, and to place contemporary research and assessments of disorganized/disoriented attachment in a new constellation, in considering the emergence and movement of this psychological construct as a product of film technologies. Through this reconfiguration we hope that, beneath the constituted classifications of attachment theory, films of an infant’s separation and reunion with their attachment figure in a laboratory setting can be recognized *as* films. Thus the living motion and affective intensity may come into view in new ways, presenting alternative ways of thinking about the interpretation of conflict and disorganization from viewed behaviour.

## Footnotes

1 See, for example, Vicky LeBeau, *Lost Angels: Psychoanalysis and Cinema* (London: Routledge, 1994), and Arthur Shimamura, *Psychocinematics: Exploring Cognition at the Movies* (Oxford: Oxford University Press, 2013).

2 See, for example, Stanley Cavell, ‘The image of the psychoanalyst in film’, in *Cavell on Film*, ed. William Rothman (New York, NY: State University of New York Press, 2005), pp. 295–304.

3 Vicky Lebeau, *Childhood and Cinema* (London: Reaktion, 2008) is attentive to scientific investments in film. For an important discussion of science films as a neglected object in film studies, see Malin Wahlberg, ‘Wonders of cinematic abstraction’, *Screen*, vol. 47, no. 3 (2006), pp. 273–89.

4 Emma Wilson, *Alain Resnais* (Manchester: Manchester University Press, 2006).

5 Lisa Cartwright, ‘“Emergencies of survival”: moral spectatorship and the “new vision of the child” in postwar child psychoanalysis’, *Journal of Visual Culture*, vol. 3, no. 1 (2004), pp. 35–49.

6 See, for example, Claire Hughes and Rory T. Devine, ‘Silent films and strange stories: theory of mind, gender and social experiences in middle childhood’, *Child Development*, vol. 84, no. 3 (2013), pp. 989–1003.

7 Monica Greco, ‘Logics of interdisciplinarity: the case of medical humanities’, in Georgina Born and Andrew Barry (eds), *Interdisciplinarity: Reconfigurations of the Social and Natural Sciences* (London: Routledge, 2013), pp. 226–46.

8 Annette Kuhn (ed.), *Little Madnesses: Winnicott, Transitional Phenomena and Cultural Experience* (London: IB Tauris, 2013).

9 See, for example, Vicky Lebeau, ‘Daddy’s cinema: femininity and mass spectatorship’, *Screen*, vol. 33, no. 3 (1992), pp. 244–58; Dimitris Eleftheriotis, ‘Early cinema as child: historical metaphor and European cinephilia in *Lumiere & Company* ’, *Screen*, vol. 46, no. 3 (2005), pp. 315–28; Kathryn Bond Stockton, *The Queer Child, or Growing Up Sideways in the Twentieth Century* (Durham, NC: Duke University Press, 2010).

10 See, for example, Miriam Steele, Howard Steele, Jordan Bate, Hannah Knafo, Michael Kinsey, Karen Bonuck, Paul Meisner and Anne Murphy, ‘Looking from the outside in: the use of video in attachment-based interventions’, *Attachment and Human Development*, vol. 16, no. 4 (2014), pp. 402–15.

11 John Bowlby and James Robertson, *Protest, Despair and Detachment* (Unpublished manuscript, 1965), Wellcome Trust Library Archive, <http://archives.wellcomelibrary.org/DServe/dserve.exe?dsqIni=Dserve.ini&dsqApp=Archive&dsqCmd=Show.tcl&dsqDb=Catalog&dsqSearch=(AltRefNo=%27pp/bow%27)&dsqPos=0> accessed 26 September 2016. The little girl’s own reaction to seeing the film is also of note here: ‘after reunion a residue of reproach larger than is suspected may remain. This was shown dramatically by Laura six months after her return home, by which time she had seemed to be her normal self. Late one evening when her parents were viewing a rough version of the film, through a mischance Laura came in to see the final sequences. When the lights went up she was found to be agitated. Suddenly she flushed, turned angrily to her mother and exclaimed, “Where *was* you all the time, Mummy, where *was* you?” Then she burst into loud crying.’ John Bowlby, ‘Pathological mourning and childhood mourning’, *Journal of the American Psychoanalytic Association*, vol. 11, no. 3 (1963), p. 524.

12 Anna Freud, ‘A two-year-old goes to hospital – scientific film by James Robertson’, *International Journal of Psycho-Analysis*, no. 34 (1953), 285. John Bowlby writes – in *A Secure Base* (London: Routledge, 1988), p. 24 – that ‘Influential though the written word may often be, it has nothing like the emotional impact of a movie’. Over the coming years, recognition of film as a medium offering something irreducible to psychological inquiry and argument would grow among research psychologists and clinicians. By the 1960s, American family therapists Ian Algers and Peter Hogan could argue that ‘videotape recording represents a technological breakthrough with the kind of significance for psychiatry that the microscope has had for biology’, in ‘The use of videotape recordings in cojoint marital therapy’, *American Journal of Psychiatry*, vol. 123, no. 11 (1967), p. 1425.

13 See Lebeau, *Childhood and Cinema*, and Karen Lury, *The Child in Film: Tears, Fears and Fairy Tales* (London: IB Tauris, 2010).

14 Mary Ainsworth and Robert Marvin, (1995) ‘On the shaping of attachment theory and research: an interview with Mary D. S. Ainsworth’, *Monographs of the Society for Research in Child Development*, vol. 6, no. 2/3 (1995), pp. 3–21.

15 Unpublished letter from Mary Ainsworth to John Bowlby, dated 30 May 1972, Wellcome Trust Library Archive, London.

16 Jude Cassidy, Robert Marvin and the MacArthur Network on Attachment in the Preschool Years, *Attachment Organisation in Pre-School Children*: *Procedures and Coding Manual* (Unpublished manuscript, 1992), p. 20. Ainsworth was a member of the MacArthur Network.

17 Everett Waters, ‘The video record’, *Comments on Strange Situation Classification* (2002), <http://www.psychology.sunysb.edu/attachment/measures/content/ss_scoring.pdf> accessed 1 September 2016.

18 Patricia Crittenden, ‘Instructions to camera operators’, appended to *The Pre-School Assessment of Attachment Coding Manual* (Miami, FL: Family Relations Institute, 1981), p. 92.

19 Russell Tracy, Michael Lamb and Mary D. Ainsworth, ‘Infant approach behavior as related to attachment’, *Child Development*, vol. 47, no. 3 (1976), p. 577.

20 Klaus Grossmann, Karin Grossmann, Franz Huber and Ulrike Wartner, ‘German children’s behavior towards their mothers at 12 months and their fathers at 18 months in Ainsworth’s Strange Situation’, *International Journal of Behavioral Development*, vol. 4, no. 2 (1981), p. 159.

21 See, for example, Phyllis Cohen and Beatrice Beebe, ‘Video feedback with a depressed mother and her infant’, *Journal of Infant, Child and Adolescent Psychotherapy*, vol. 2, no. 3 (2002), pp. 1–55, and David Shemmings and Yvonne Shemmings, *Assessing Disorganised Attachment Behaviour in Children* (London: JKP, 2014).

22 Steele, et al., ‘Looking from the outside in’, p. 412.

23 Ellen Moss, G. Tarabulsy, Rachèle St-Georges, Karine Dubois-Comtois and Vanessa Lecompte, ‘Video-feedback intervention with maltreating parents and their children’, *Attachment and Human Development*, vol. 16, no. 4 (2014), pp. 333–34; see also Marina Zelenko and Anne Benham, ‘Videotaping as a therapeutic tool in psychodymamic infant psychotherapy’, *Infant Mental Health*, vol. 21, no. 3 (2000), pp. 192–203. Steele and colleagues observe that film adds something irreducible within attachment-based interventions, since ‘video enhances the therapeutic alliance, facilitates a reflective stance in the parent, and consolidates clinical gains by focusing the parents’ attention on specific attachment related behaviors that they are asked to translate into words, thereby helping to “metabolize” what are often difficult to process thoughts and feelings.’ Steele, et al., ‘Looking from the outside in’, p. 409.

24 Femmie Juffer, Marian J. Bakermans-Kranenburg and Marinus H. van Ijzendoorn, *Promoting Positive Parenting: An Attachment-Based Intervention* (London: Routledge, 2007).

25 Lebeau, *Childhood and Cinema*, p. 19.

26 Mary Ainsworth, ‘Attachments and other affectional bonds across the life cycle’, in C. M. Parkes, J. Stevenson-Hinde and P. Marris (eds), *Attachment Across the Life Cycle* (London: Routledge, 1991), pp. 33–51; Mary Main, ‘Cross-cultural studies of attachment organisation’, *Human Development*, no. 33 (1990), p. 4861.

27 Alan Sroufe and Everett Waters, ‘Attachment as an organizational construct’, *Child Development*, no. 48 (1977), pp. 1184–99.

28 Mary D. Ainsworth, ‘Attachment and dependency: a comparison’, in Jacob L. Gewirtz (ed.), *Attachment and Dependency* (Washington, DC: Wiley, 1972), pp. 97–138.

29 For a review, see Alan Sroufe, Elizabeth Carlson, Alissa Levy and Byron Egeland, ‘Implications of attachment theory for developmental psychopathology’, *Development and Psychopathology*, no. 11 (1999), pp. 1–13.

30 See, for example, Elizabeth A. Carlson, Alan Sroufe and Byron Egeland, ‘The construction of experience: a longitudinal study of representation and behavior’, *Child Development*, no. 75 (2004), pp. 66–83.

31 Mary Main, ‘Analysis of a peculiar form of reunion behaviour seen in some day-care children’, in Robert Webb (ed.), *Social Development in Childhood* (Baltimore, MD: Johns Hopkins University Press, 1977), p. 69.

32 Patricia Crittenden, ‘Mother and infant patterns of attachment’ (Unpublished PhD dissertation: University of Virginia, 1983).

33 Selma Fraiberg, ‘Pathological defences in infancy’, *Psychoanalytic Quarterly*, no. 51 (1981), pp. 612–35.

34 Mary Main and Donna Weston, ‘The quality of the toddler’s relationship to mother and to father: related to conflict behavior and the readiness to establish new relationships’, *Child Development*, vol. 52, no. 3 (1981), p. 939.

35 Mark B. Hansen, ‘The time of affect, or bearing witness to life’, *Critical Inquiry*, no. 30 (2004), pp. 584–626; Laura Mulvey, *Death 24x a Second* (London: Reaktion Books, 2006).

36 Main and Solomon, ‘Procedures for identifying’, p. 144.

37 Mary Main and Jackolyn Stadtman, ‘Infant response to rejection of physical contact by the mother’, *Journal of the American Academy of Child Psychiatry*, vol. 20, no. 2 (1981), p. 297.

38 Mary Main and Erik Hesse, personal communication, 8 January 2013.

39 Main and Solomon, ‘Procedures for identifying’, p. 143.

40 Ibid., p. 133.

41 Robbie Duschinsky, ‘The emergence of the “disorganised/disoriented (D) attachment” classification, 1979–1982’, *History of Psychology*, vol. 18, no. 1 (2015), pp. 32–46.

42 Mary Ainsworth, unpublished letter to Bowlby, dated 14 February 1985, Wellcome Trust Library Archive.

43 Pasco Fearon, Marian J. Bakermans-Kranenburg, Marinus H. Van Ijzendoorn, Anne-Marie Lapsley and Glenn Roisman, ‘The significance of insecure attachment and disorganization in the development of children’s externalizing behavior’, *Child Development*, vol. 81, no. 2 (2010), pp. 435–56.

44 Helen MacDonald, Marjorie Beeghly, Wanda Grant-Knight, Marylin Augustyn, Ryan Woods, Howard Cabral, et al., ‘Longitudinal association between infant disorganized attachment and childhood posttraumatic stress symptoms’, *Development and Psychopathology*, no. 20 (2008), pp. 493–508; Michelle Enlow, Byron Egeland, Elizabeth Carlson, Emily Blood and Rosalind Wright, ‘Mother–infant attachment and the intergenerational transmission of posttraumatic stress disorder’, *Development and Psychopathology*, no. 26 (2014), pp. 41–65.

45 Attachment Videos Library, <http://www.psychology.sunysb.edu/attachment/video_contents/videos_index_2010_kg2_infant_script.html> accessed 1 September 2016.

46 Mary Main and Erik Hesse, ‘Parents’ unresolved traumatic experiences are related to infant disorganized attachment status’, in M. T. Greenberg, D. Cicchetti and E. M. Cummings (eds), *Attachment in the Preschool Years* (Chicago, IL: University of Chicago Press, 1990), p. 163.

47 Judith Solomon and Carol George, ‘Defining the caregiving systems: toward a theory of caregiving’, *Infant Mental Health Journal*, no. 17 (1996), pp. 3–17. See also Karlen Lyons-Ruth and Eda Spielman, ‘Disorganized infant attachment strategies and helpless-fearful profiles of parenting’, *Infant Mental Health Journal*, vol. 25, no. 4 (2004), pp. 318–35.

48 Mary Main, Erik Hesse and Siegfried Hesse, ‘Attachment theory and research: overview with suggested applications to child custody’, *Family Court Review*, vol. 49, no. 3 (2011), p. 441.

49 To take but one example, Ross D. Parke and Alison Clarke-Stewart in *Social Development* (Hoboken, NJ: John Wiley, 2010) explain that children classed as having ‘insecure-disorganised attachment (Type D) […] seem to be apprehensive and fearful of their attachment figure’. More generally, for the psy-disciplines, see Nikolas Rose, *Inventing Our Selves: Psychology, Power and Personhood* (Cambridge: Cambridge University Press, 1989).

50 Erik Hesse and Mary Main, ‘Frightened, threatening and dissociative parental behaviour in low-risk samples: description, discussion and interpretations’, *Development and Psychopathology*, vol. 18, no. 2 (2006), pp. 309–43.

51 The same retrospective assessment of Main’s texts has been made by Elena Padrón, Elizabeth Carlson and Alan Sroufe, ‘Frightened versus not frightened disorganized infant attachment’, *American Journal of Orthopsychiatry*, vol. 84, no. 2 (2014), pp. 201–8.

52 See, for example, Judith Mayne, *Cinema and Spectatorship* (London: Routledge, 1993), and Rob Lapsley, ‘Cinema, the impossible, and a psychoanalysis to come’, *Screen*, vol. 50, no. 1 (2009), pp. 14–24.

53 Erving Goffman, *Frame Analysis* (Boston, MA: Northeastern University Press, 1974), p. 346.

54 Alan Sroufe, Strange Situation training, University of Minnesota, 12 June 2014. Permission was given for this remark to be cited.

55 Main and Solomon, ‘Procedures for identifying’, p. 147.

56 Ibid.

57 Elizabeth Cowie, *Recording Reality, Desiring the Real* (Minnesota, MN: University of Minnesota Press, 2011), p. 9. See also Kaja Silverman, *The Threshold of the Visible World* (London: Routledge, 1996), and Tom Gunning, ‘Moving away from the index: cinema and the impression of reality’, *Differences*, vol. 18, no. 1 (2007), pp. 29–52.

58 See, for example, Erica Burman, *Deconstructing Developmental Psychology*, 2nd edn (London: Routledge, 2007), and Steve Brown and Paul Stenner, *Psychology without Foundations* (London: Sage, 2009).

59 Robbie Duschinsky, Monica Greco and Judith Solomon, ‘The politics of attachment: lines of flight’, *Theory, Culture and Society*, vol. 32, no. 7/8 (2015), pp. 173–95.

60 Main and Solomon, ‘Procedures for identifying’, p. 156.

61 Karlen Lyons-Ruth, Jean-Francois.Bureau, M. Ann Easterbrooks, Ingrid Obsuth and Kate Hennighausen, ‘Parsing the construct of maternal insensitivity: distinct longitudinal pathways associated with early maternal withdrawal’, *Attachment and Human Development*, vol. 15, no. 5/6, (2013), pp. 562–82; see also Andrea Landini, Clark Baim, Marta Hart and Sophie Landa, *Danger, Development and Adaptation* (Hook: Waterside, 2015).

62 Elizabeth Meins, Charles Fernyhough, Bronia Arnott, Lucia Vittorini, Michelle Turner, Susan. R. Leekam and Katheryn Parkinson, ‘Individual differences in infants’ joint attention behaviors with mother and a new social partner’, *Infancy*, vol. 16, no. 6 (2011), p. 606.

63 Bryan S. Turner, *Regulating Bodies: Essays in Medical Sociology* (London: Routledge, 1992), p. 70.

64 See, for example, Ann Oakley, *Subject Women* (Oxford: Martin Robertson, 1971), and Julia Krane, Linda Davies, Rosemary Carlton and Meghan Mulcahy, ‘The clock starts now: feminism, mothering and attachment theory in child protection practice’, in Brid Featherstone, Carol-Ann Hooper, Jonathan Scourfield and Julie Taylor (eds), *Gender and Child Welfare in Society* (Oxford: Blackwell, 2010), pp. 149–72.

65 Gilles Deleuze and Félix Guattari, *Anti-Oedipus*, trans. Robert Hurley (New York, NY: Continuum, 1984 [1971]); Bringing together Deleuze with developmental psychology is in line with calls to use and develop Deleuze’s ideas about childhood. For example, Anna Hickey-Moody has urged recognition that ‘Deleuze’s writings on children offer possibilities for rethinking the process of growing up’, and also that ‘Deleuze’s theory of childhood affectivity is inherently conservative to the extent that it romanticizes the childhood state’. Anna Hickey-Moody, ‘Deleuze’s children’, *Educational Philosophy and Theory*, vol. 45, no. 3 (2013), pp. 284, 281.

66 Robbie Duschinsky, Monica Greco and Judith Solomon, ‘Wait up! Attachment and sovereign power’, *International Journal of Politics, Culture and Society*, vol. 28, no. 3 (2015), pp. 223–42.

67 Gilles Deleuze, *Cinema 2: The Movement-Image*, trans. Hugh Tomlinson and Robert Galeta (London: Bloomsbury, 1989 [1985]), p. 124.

68 Kurt Lewin, *Conceptual Representation and Measurement of Psychological Forces* (Durham, NC: Duke University Press, 1938), p. 188.

69 Deleuze, *Cinema 2*, p. 124.

70 Gilles Deleuze, *Bergsonism*, trans. Hugh Tomlinson and Barbara Habberjam (New York, NY: Zone, 1988 [1966]), p. 19 (parentheses suppressed).

71 Deleuze, *Cinema 2*, p. 134.

72 Benedict Spinoza, *Ethics*, trans. Edwin Curley (London: Penguin, 1994 [1677]) Part III, Prop. 17.

73 Gilles Deleuze, *Expressionism in Philosophy*, trans. Martin Joghin (New York, NY: Zone, 1989 [1968]), pp. 386–87, n. 23, and ‘On Gilbert Simondon’, trans. Michael Taormina (New York, NY: Semiotext(e), 2004 [1966]). Cf. Pierre Macherey, ‘The encounter with Spinoza’, in Paul Patton (ed.), *Deleuze: A Critical Reader* (Oxford: Blackwell, 1996), pp. 139–61.

74 Deleuze, *Cinema 2*, p. 135.

75 Ibid., p. 209.

76 Main and Solomon, ‘Procedures for identifying’, p. 136.

77 See, for example, R. Chris Fraley, Glenn I. Roisman, Cathryn Booth-LaForce, Margaret T. Owen and Ashley S. Holland, ‘Interpersonal and genetic origins of adult attachment styles: a longitudinal study from infancy to early adulthood’, *Journal of Personality and Social Psychology*, vol. 104, no. 5 (2013), pp. 817–38; web-based supplement, Part C: ‘The second dimension, *Angry and resistant strategies*, represents variability in the amount of overt conflict and anger the child expressed toward the caregiver during the strange situation, and was computed as the average of the ratings for the following interactive coding scales: Resistance Episode 5, Resistance Episode 8, and Disorganization’.

78 Lewin, *Conceptual Representation and Measurement of Psychological Forces*, p. 188.

79 Michel Foucault, *Archaeology of Knowledge* (London: Routledge, 1972 [1969]), p. 75 (translation emended).

